# The protective effect of recombinant *Lactococcus lactis* oral vaccine on a *Clostridium difficile*-infected animal model

**DOI:** 10.1186/1471-230X-13-117

**Published:** 2013-07-17

**Authors:** Xiao-qiang Yang, Ya-gang Zhao, Xue-qing Chen, Bo Jiang, Da-yong Sun

**Affiliations:** 1 Department of Gastroenterology, General Hospital of Guangzhou Military Command of People’s Liberation Army, Guangzhou 510010, Guangdong, China; 2 Department of Gastroenterology, The First Affiliated Hospital of Guangzhou Medical University, No. 151, Yanjiang Road, Guangzhou 510120, Guangdong, China; 3 Department of Gastroenterology, Nanfang Hospital, Southern Medical University, Guangzhou 510515, China

**Keywords:** Clostridium difficile, Recombinant *Lactococcus lactis*, Secreted-protein plasmid, Membrane-anchored plasmid, Golden hamsters

## Abstract

**Background:**

Oral immunization with vaccines may be an effective strategy for prevention of *Clostridium difficile* infection (CDI). However, application of previously developed vaccines for preventing CDI has been limited due to various reasons. Here, we developed a recombinant *Lactococcus lactis* oral vaccine and evaluated its effect on a *C. difficile-*infected animal model established in golden hamsters in attempt to provide an alternative strategy for CDI prevention.

**Methods:**

Recombinant *L. lactis* vaccine was developed using the pTRKH2 plasmid, a high-copy-number *Escherichia coli*-*L.* shuttle vector: 1) *L. lactis* expressing secreted proteins was constructed with recombinant pTRKH2 (secreted-protein plasmid) carrying the Usp45 signal peptide (SPUsp45), nontoxic adjuvanted tetanus toxin fragment C (TETC), and 14 of the 38 C-terminal repeats (14CDTA) of nontoxic *C. difficile* toxin A (TcdA); and 2) *L. lactis* expressing secreted and membrane proteins was constructed with recombinant pTRKH2 (membrane-anchored plasmid) carrying SPUsp45, TETC, 14CDTA, and the cell wall-anchored sequence of protein M6 (cwaM6). Then, 32 male Syrian golden hamsters were randomly divided into 4 groups (n = 8 each) for gavage of normal saline (blank control) and *L. lactis* carrying the empty shuttle vector, secreted-protein plasmid, and membrane-anchored plasmid, respectively. After 1-week gavage of clindamycin, the animals were administered with *C. difficile* spore suspension. General symptoms and intestinal pathological changes of the animals were examined by naked eye and microscopy, respectively. Protein levels of anti-TcdA IgG/IgA antibodies in intestinal tissue and fluid were analyzed by enzyme-linked immunosorbent assay (ELISA). A cell culture cytotoxicity neutralization assay was done by TcdA treatment with or without anti-TcdA serum pre-incubation or treatment. Apoptosis of intestinal epithelial cells was examined by flow cytometry (FL) assay. Expression of mucosal inflammatory cytokines in the animals was detected by polymer chain reaction (PCR) assay.

**Results:**

After the *C. difficile* challenge, the animals of control group had severe diarrhea symptoms on day 1 and all died on day 4, indicating that the CDI animal model was established in hamster. Of the 3 immunization groups, secreted-protein and membrane-anchored plasmid groups had significantly lower mortalities, body weight decreases, and pathological scores, with higher survival rate/time than the empty plasmid group (P < 0.05). The tilter of IgG antibody directed against TcdA was significantly higher in serum and intestinal fluid of secreted-protein and membrane-anchored plasmid groups than in the empty plasmid group (P < 0.05) while the corresponding titer of IgA antibody directed against TcdA had no substantial differences (P > 0.05). The anti-TcdA serum of membrane-anchored plasmid group neutralized the cytotoxicity of 200 ng/ml TcdA with the best protective effect achieved by anti-TcdA serum pre-incubation. The incidences of TcdA-induced death and apoptosis of intestinal epithelial cells were significantly reduced by cell pre-incubation or treatment with anti-TcdA serum of membrane-anchored plasmid group (P < 0.05). MCP-1, ICAM-1, IL-6, and Gro-1 mRNA expression levels were the lowest in cecum tissue of the membrane-anchored groups compared to the other groups.

**Conclusion:**

Recombinant *L. lactis* live vaccine is effective for preventing CDI in the hamster model, thus providing an alternative for immunization of *C. difficile-*associated diseases.

## Background

*Clostridium difficile* is one of the most important pathogens in nosocomial infections. *C. difficile* infection (CDI) causes 10–20% of antibiotic-associated diarrhea, 75% of antibiotic-associated colitis, and nearly 100% of pseudomembranous colitis in hospitals (referred to as *C. difficile*-associated diseases, CDADs), leading to billions of dollars in economic losses worldwide every year [1]. In general, CDADs occur mainly in people with long-term use of antibiotics, the use of anticancer drugs, long-term hospitalization or immune defects, especially those with a decline in immune function or the elderly [1]. With the development of medical industry and the increasing use of antibiotics, the rate of CDI has been substantially increased in China [2]. Effective strategies are urgently needed for CDI prevention in the high-risk population of CDADs.

Due to antibiotic resistance and inherent physiological factors of the pathogen, antibiotic treatment of CDI can be challenging while oral immunization with vaccines is generally considered to be an important pathway for CDI prevention [1]. Vaccine development involves the establishment of an appropriate animal model and further evaluation of the efficacy and safety of the vaccine. In previous research of *C. difficile* vaccines, Kink *et al.*[3] successfully established CDI model in hamster by intragastric gavage of 10^5^CFU *C. difficile* 18–24 h after subcutaneous injection of clindamycin (CLDM, 1 mg per 100 g body weight), and Torres *et al*. [4] administered the animals with 10^5^CFU *C. difficile* 3-h after CLDM gavage (0.5 mg per 100 g body weight). In general, golden hamster is considered ideal for establishing CDI model, because *C. difficile*-produced toxins can be neutralized by anti-*C. difficile* antibodies and CLDM-induced colitis model can be used as animal model of human CDIs.

Regarding the development of vaccines for *C. difficile*, great research progress has been made over the last two decades [1]. For example, Torres *et al.*[4] reported that formalin-inactivated *C. difficile* culture filtrate has a protective effect on hamsters by nasal, peritoneal, or subcutaneous administration. Ryan *et al.*[5] and Ward *et al.*[6] developed recombinant vaccines for *C. difficile* by engineering a plasmid to express recombinant toxin A proteins from the nontoxic C-terminal receptor binding region of *C. difficile* toxin A (TcdA) covalently bonded to polysaccharide of other bacterium, and then introducing this plasmid into attenuated *Salmonella typhimuriu* or *Vibrio cholerae*. However, the application of previously developed vaccines for *C. difficile* has been limited for various reasons: 1) Vaccines for passive immunization of CDI are thought expensive and inconvenient in storage and transport; 2) Attenuated vaccines are commonly treated with formalin to inactivate the antigen or given with adjuvant, sometimes through invasive routes of immunization such as subcutaneous and intraperitoneal injection, thus are not easily accepted by the patients. 3) Recombinant vaccines that are carried by attenuated *S. typhimuriu* or *V. cholerae* are of concern in terms of biosafety [1].

*Lactococcus lactis* is a harmless food industry bacterium that has been used extensively for producing a variety of peptides, proteins, and oral vaccines. As compared to the vaccine carrier *E. coli*, *L. lactis* can be a superior alternative because it produces less protease with no endotoxin [7]. In the literature, Dieye *et al*. [8] designed a protein-targeting system for lactic acid bacteria and found that the *L. lactis* expression system constructed with the P59 promoter and Usp45 single peptide (SPUsp45) was capable of expression and extracellular secretion of target nuclease while the expression system constructed with P59, Usp45, and the cell wall-anchored sequence of protein M6 (cwaM6) was capable of extracellular secretion of the nuclease as well as anchoring it onto the cell wall of *L. lactis* and *Bacterium lacticum.* In a following study, Ribeiro *et al.*[9] expressed a fusion protein containing the *Brucella abortus* antigen L7/L12 and the *Streptococcus pyogenes* cwaM6 in *L. lactis*, which allowed the antigen anchored on the cell surface, thus improving its antigenicity. These authors further developed a food-grade live vaccine for immunization of *B. abortus*, which showed a protective effect on mice under laboratory conditions [9]. In addition, Mannam *et al.*[10] made a mucosal vaccine from live, recombinant *L. lactis*, which protected mice against pharyngeal infection with *S. pyogenes*. Together these researches have provided a completely new direction for development of vaccines, especially live probiotic preparations for oral immunization.

Despite potential advantages of *L. lactis* as the carrier of live vaccine, no studies have been reported on oral immunization with *L. lactis* live vaccines for preventing CDI till date. Whether vaccines made from live, recombinant *L. lactis* are effective for preventing CDI remains unclear. Previously, we constructed a gene expression system in *L. lactis* based on the work by Dieye *et al.*[8]. In the present study, we modified the gene expression system to develop recombinant *L. lactis* live vaccine for *C. difficile*, and then vaccinated a CDI animal model of golden hamsters by oral immunization. Pathological and immunological parameters of the animals were assayed under laboratory conditions. Results were used to evaluate the effect of recombinant *L. lactis* live vaccine for preventing CDI.

## Methods

### Plasmid, *C. difficile* culture, cell lines

The pTRKH2 plasmid [11], a high-copy-number *E. coli*-*L. lactis* shuttle vector, was kindly provided by Klanenhammer TR (Department of Food Science, North Carolina State University). *C. difficile* VPI10463 was purchased from Lanzhou Institute of Biological Products (Lanzhou, China). The pathogen was cultured in Brain-Heart Infusion medium (BHI) (Difco) containing 5 mg/ml yeast extract and 0.1% L-cysteine at 37°C for 72 h in an anaerobic chamber (Coy Laboratory Products). CHO-K1 and T84 cells were purchased from the Institute of Basic Medical Sciences, Chinese Academy of Medical Sciences (Beijing, China). The two cell lines were respectively cultured in F12 and DMEM-F12 media (Gibco) containing 10% heat-inactivated fetal bovine serum, 100 U/ml penicillin, and 100 μg/ml streptomycin at 37°C in a CO_2_ incubator (5% CO_2_, saturated humidity).

### Preparation of recombinant *L. lactis* live vaccine and *C. difficile* spore suspension

Recombinant *L. lactis* live vaccine was prepared using the shuttle vector pTRKH2 as previously reported by Yang *et al*. [12,13]. A slight modification was that the nontoxic tetanus toxin fragment C (TETC) was inserted to the *L. lactis* expression system as a biological adjuvant. That is, *L. lactis* expressing secreted proteins was constructed with recombinant pTRKH2 (secreted-protein plasmid) carrying the secretory signal peptide SPUsp45, nontoxic adjuvanted TETC, and 14 of the 38 C-terminal repeats (14CDTA) of TcdA; and *L. lactis* expressing secreted and membrane proteins was constructed with recombinant pTRKH2 (membrane-anchored plasmid) carrying SPUsp45, TETC, 14CDTA, and the cell wall-anchored sequence cwaM6. *L. lactis* cell suspension (5 × 10^9^CFU/ml) was prepared in 0.2M sodium bicarbonate containing 5% casein hydrolyzate and 0.5% glucose.

*C. difficile* was induced on BHIS agar as described previously [14]. Briefly, the overnight culture broth of *C. difficile* in BHIS medium was diluted in fresh BHIS medium to an optical density (OD, 600 nm) of 0.2. Then, 150 ml of diluted culture suspension was spread on 5 ml of BHIS agar dispensed in each well of a 6-well tissue culture dish, followed by anaerobic incubation at 37°C for 4–7 d. To examine the colony formation from *C. difficile* spores, samples were taken from the plates containing a mixture of spores and vegetative cells and then resuspended in BHIS medium. The suspension was heated to 60°C for 20 min to kill vegetative cells and then cooled, diluted and plated onto BHIS medium. For use in germination assays, *C. difficile* spores were purified using the method of Akoachere *et al.*[15] withminor modifications. The spore-vegetative cell mixture was harvested by flooding each well of the 6-well dish with ice-cold sterile water. After 5 washes with ice-cold water, the mixture of spores and vegetative cells were resuspended in 20%w/v HistoDenz (Sigma, St. Louis, MO, USA), layered onto a 50%w/v HistoDenz solution in a centrifuge tube, and then centrifuged at 15,0007× *g* for 15 min to separate spores from vegetative cells. The purified spores, collected at the bottom of the centrifuge tube, were washed twice with ice-cold water to remove traces of HistoDenz and then resuspended in distilled water.

### Animal experiment

#### ***Oral immunization with L. lactis vaccine***

Thirty-two male Syrian golden hamsters with a weight of 100–130 g were purchased from the Slac Laboratory Animal Co., Ltd. (Shanghai, China). The golden hamsters were randomly divided into 4 groups (n = 8 each) for gastric perfusion of 1 ml suspension of normal saline (control) and *L. lactis* carrying the empty shuttle vector, secreted-protein plasmid, and membrane-anchored plasmid, respectively (on days 0, 1, 2, 7, 14, and 23). Intestinal flora in the animals was examined on days 7 and 14 prior to gastric perfusion. Mortality, diarrhea incidence, weight change from before to after the challenge, and survival time of the animals were examined and determined for evaluating the effect of recombinant *L. lactis* vaccine on the CDI animals model. The animal experiment was approved by the Ethics committee of General Hospital of Guangzhou Military Command of People’s Liberation Army.

#### ***C. difficile challenge experiment***

**Figure 1:**
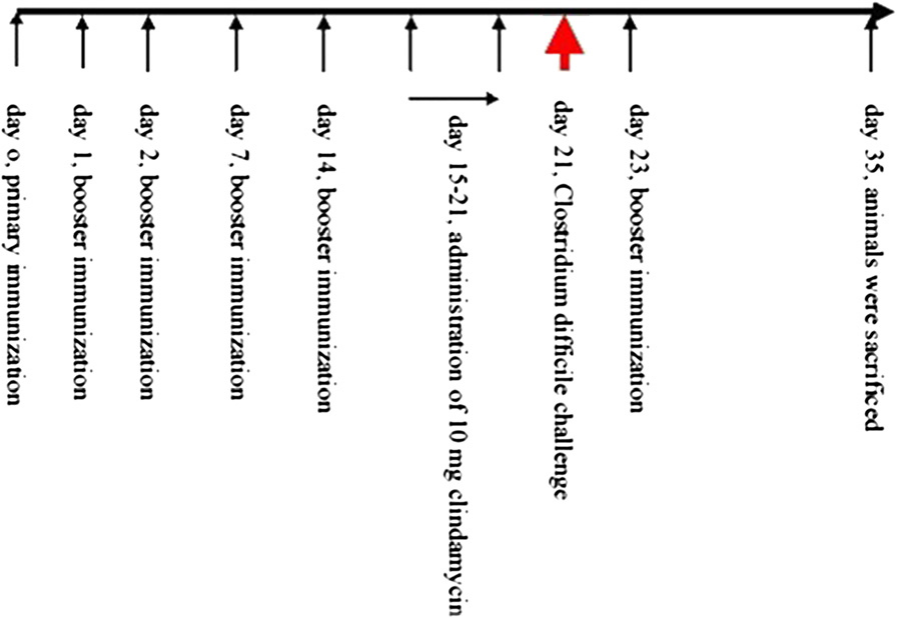
**Schematic diagram of *Clostridium difficile *challenge experiment.**

From day 15, 10 g of CLDM (clindamycin hydrochloride, North China Pharmaceutical Co., Ltd, Shijiazhuang, China) was administered to the animals by gavage once daily for one week. Then, intestinal flora of the animals was analyzed, and *C. difficile* was cultured and isolated to determine potential imbalance in the intestinal flora and the incidence of CDI. Each animal was weighed and then exposed to 4 × 10^5^CFU *C. difficile* 4 h after the last administration of CLDM on day 21. The animals were observed every 4 h for 72 h. Feces samples were taken for cultivation and isolation of *C. difficile* and *L. lactis*. Thereafter, the animals were observed 4 times a day on the feces, perianal area, activity, fur morphology, and gloss and mental status. The animals were scored based on the observations as follows [3,4]: 0, normal; 1, light stool, normal activity; 2, wet perianal and tail, close to normal activity; 3, less activity but respond to stimuli; soft abdomen; and 4, wet tail, claws and lower abdomen; curled; no activity; soft abdomen; balance impaired; fur shrug. Animals were killed when the score was 4, and the surviving animals were sacrificed at the end of the experiment on day 35, i.e., 14 days after *Clostridium* exposure (Figure 1).

#### ***Serum, intestinal tissue, and intestinal fluid specimen preparation***

The animals were treated with ether for mild anesthesia before sacrificed. Skin infection was done with alcohol. The thoracic cavity was cut and opened with scissors. Blood was taken from the heart through a fine needle and then centrifuged at 13000 g, 4°C for 15 min. The supernatant serum was collected and stored at -70°C prior to serum enzyme-linked immunosorbent assay **(**ELISA). For preparation of intestinal specimens, the intestinal tract was taken from the animals, with surrounding blood vessels and adipose tissues carefully removed. The remaining material was washed with pre-cooled phosphate-buffered saline (PBS). Then, 100 mg of intestinal tissue was weighed into a 1.5-ml microcentrifuge tube containing 0.5 ml of TE buffer with the proteinase inhibitor Cocktail (Roche, USA). Protein components were extracted by homogenization and subsequent centrifugation (13000 g, 4°C, 15 min), with the supernatant collected and stored at -70°C prior to use. Finally, 100 mg of ileum to colon tissue was weighted into a 1.5-ml microcentrifuge containing 0.5 ml of PBS buffer with trypsin inhibitor. The ileum to colon tissue was cut into pieces and moderately stirred for 30 min, then incubated at 4°C for 10 h. After centrifugation (13000 g, 4°C for 15 min), the supernatant (intestinal mucus) was collected and stored at -70°C prior to use.

#### ***Pathological analysis and scoring***

The animals of different treatments were dissected, with abdominal and intestinal lesions examined by naked eye. Part of the cecum and colon tissues was taken and cut along the longitudinal axis. After the intestinal contents were washed with 4°C normal saline, 100 mg of intestinal mucosal tissue was weighed and immediately homogenized for cultivation and isolation of *C difficile* and *L. lactis*. Intestinal total RNA and total protein, cytoplasmic protein and nuclear protein of intestinal epithelial cells were extracted from 200 mg of intestinal mucosal tissue. Pathological changes in the intestine were observed under a stereoscope and a dissecting microscope (Olympus SZX7, Tokyo, Japan). Then, the tissues were fixed in 10% neutral formalin, embedded in paraffin, HE-stained, and observed under a light microscope (Olympus BX40, Tokyo, Japan). The criteria for the macropathological score and histopathological score are shown in Tables 1 and 2, respectively.

**Table 1:** **Criteria for macropathological scoring of *Clostridium difficile*-infected hamsters**

**Gross macropathology**	**Score**
Mucosal lesion	
No	0
Local congestion, edema, non-erosive	1
1-2 erosion	Additional 1–2 points
>2 erosions	Additional 3 points
One ulcer	Additional 4 points
>2 ulcers	Additional 5 points
Complicated with hemorrhage	Additional 1–3 points

**Table 2:** **Criteria of histopathological scoring of *Clostridium difficile*-infected hamsters**

**Histological changes**	**Score**
Inflammatory cell infiltration	
No	0
Mild	1
Medium	2
Severe	3
Mucosal defect	
No	0
Local	1
Extensive	2
Epidermal cell necrosis, Vacuolization	
No	0
Mild	1
Medium	2
Severe	3
Recess damage	
No	0
Mild	1
Medium	2
Severe	3
Mucosal hemorrhage	
No	0
Mild	1
Medium	2
Severe	3

#### ***Evaluation index system and statistical analyses***

The effect of recombinant *L. lactis* on *C. difficile*-infected animals was evaluated using the following indices: mortality, diarrhea incidence, weight changes from before to after *C. difficile* challenge, and survival time of the infected animals. Mortality and diarrhea incidence data were analyzed by Pearson’s chi-square test. P ≤ 0.05 was considered statistically significant. Weight change and pathological score data were as the mean ± standard deviation and analyzed using one-way ANOVA SNK test. The variance nonhomogeneity was tested by rank sum test, and P ≤ 0.05 considered statistically significant. Survival time data were analyzed using the Kaplan-Meier method. SPSS 13.0 was used for statistical analyses.

### Immunological evaluation

The tilters of anti-*C. difficile* toxin A (TcdA) antibodies in serum, intestinal tissue, and intestinal fluid specimens were determined using an anti-TcdA hamster IgG/IgA ELISA test kit (Adlitteram Diagnostic Laboratories) following the manufacturer’s instructions. Results were compared among different treatments using single-factor analysis of variance (ANOVA). Data were checked for normal distribution prior to statistical analysis and log-transformed when necessary.

### Cell culture cytotoxicity neutralization assay

The antagonism of anti-TcdA antibodies produced in hamster serum of the membrane-anchored plasmid group was evaluated by a cell culture cytotoxicity neutralization assay [16]. Briefly, CHO-K1 cells in the exponential growth stage were harvested and digested with 0.25% trypsin, then inoculated to sterile, dry coverslips pre-treated with anti-degreasing agent (poly-L-lysine) in a 6-well plate (~10^5^ cells/well). The overnight cultures were randomly divided into 4 groups for treatment with no-TcdA (normal control), TcdA (Calbiochem, Germany), TcdA following anti-TcdA serum pre-incubation, and TcdA with anti-TcdA serum treatment. Except for the control group, to the other 3 groups was added 100 μl of TcdA in PBS (2 μg/ml). To the anti-TcdA serum pre-incubation group, TcdA was added after 30 min pre-incubation with 100 μl of 0.22 μm-filtered serum; and to the anti-TcdA serum treatment group, TcdA was added simultaneously with 100 μl of 0.22 μm-filtered serum. The reaction in all treatments was terminated after 12-hrs incubation. The coverslips were washed 4 times with Hank’s buffered salt solution and fixed with freshly made fixative (methanol-glacial acetic acid, 3:1, v/v) for 5 min, followed by air-drying and 15 min Giemsa stain. The stained coverslips were air-dried, embedded with resin, and then examined under a light microscope. Samples with 50% or more cell rounding were considered positive if the cytotoxicity was neutralized by TcdA antitoxin [16].

### Flow cytometry assay

To examine the effect of hamster serum from the membrane-anchored plasmid group on TcdA-induced intestinal epithelial cell apoptosis, T84 cells in the exponential growth stage were harvested and prepared in 4 groups: [1] normal control, cells received no reagent; [2] TcdA treatment, cells received 100 μl of TcdA (final conc. 200 ng/ml) followed by 100 μl of PBS 1-h later; [3] anti-TcdA serum pre-incubation, cells received 100 μl of anti-TcdA serum followed by 100 μl of TcdA (final conc. 200 ng/ml) 1-h later; and [4] anti-TcdA serum treatment, cells received 100 μl of TcdA (final conc. 200 ng/ml) followed by 100 μl of anti-TcdA serum 30-min later. After 1-h treatment, ~5 × 10^5^ cells were harvested from each group by centrifugation (1000 g, 4°C, 1min) and then stained with Annexin V-FITC and propidium iodide (PI). The FITC and PI signals from stained cells were detected using flow cytometry. Intestinal epithelial cell apoptosis was examined by analyzing the binding rate of Annexin V-FITC and cells, and dead or dying cells were scored by PI signals.

### Polymerase chain reaction (PCR) assay

Total RNA was extracted from cecum tissue using Trizol reagent (Invitrogen, CA, USA), and then reverse-transcribed into cDNA using PCR techniques. The obtained cDNA served as template for PCR assay of monocyte chemoattractant protein-1 (MCP-1), intercellular adhesion molecule-1 (ICAM-1), interleukin-6 (IL-6), antiproliferative protein (APRO) and growth-regulated protein-1 (Gro-1) expression in cecum tissue using specific primers (Table 3). The 50 μl PCR reaction contained 1 μl of forward primer (20 μm), 1 μl of reverse primer (20 μm), 2 μl of cDNA template, 5 μl of 10x Pfu PCR buffer, 4 μl of dNTP mix (10 mM each), 0.5 μl of *Pfu* (5 U/μl), and double-distilled H_2_O to make up the volume. PCR amplification was done on a thermal cycler (Biometra UNO II, Göttingen, German) by 30 cycles of denaturation at 94°C for 45 sec, annealing at 58°C for 45 sec, and extension at 72°C for 45 sec, followed by a final extension step at 72°C for 10 min. PCR products were electrophoresed on 2% agarose gel, post-stained with Goldview DNA stain (Geneshun Biotech Ltd, Guangzhou, China), and visualized using a gel imaging system (Vilber Lourmat, France). Gel images were taken using a CCD digital camera. The integral optical density of DNA bands was calculated from the area and the intensity of corresponding electrophoretic bands using Gel-Pro Analyzer 4.0 software (Media Cybernetics, USA). The mRNA expression levels of target genes were calculated from the optical density of the sample relative to that of APRO (internal control).

**Table 3:** Primers designed for targeting the mucosal inflammatory cytokine-encoding genes in hamster model

**Gene**	**Accession no.**	**5′ to 3′ primer sequence**	**Amplicon size**
Gro-1	NM_008176	S: GCCACCCGCTCGCTTCTCTG	282bp
		AS: TTACTTGGGGACACCTTTTA	
APRO	X15267	S: GGAAGGCCGTGGTGCTGATGG	412bp
		AS: CCGAAGGAGAAGGGGGAGATG	
ICAM-1	NM_010493	S: GGGCACCCAGCAGAAGTTG	512bp
		AS: CCAGCCGAGGACCATACAGC	
IL-6	NM_031268	S: TTGGGACTGATGCTGGTGACA	205bp
		AS: GTGCATCATCGTTGTTCATACAATC	
MCP-1	NM_012333	S: CCCCACTCACCTGCTGCTACT	352bp
		AS: CACTGTCACACTGGTCACTCCTAC	

S and AS denote the sense and antisense primers, respectively.

## Results

### General symptoms of *C. difficile-*infected hamsters

**Figure 2:**
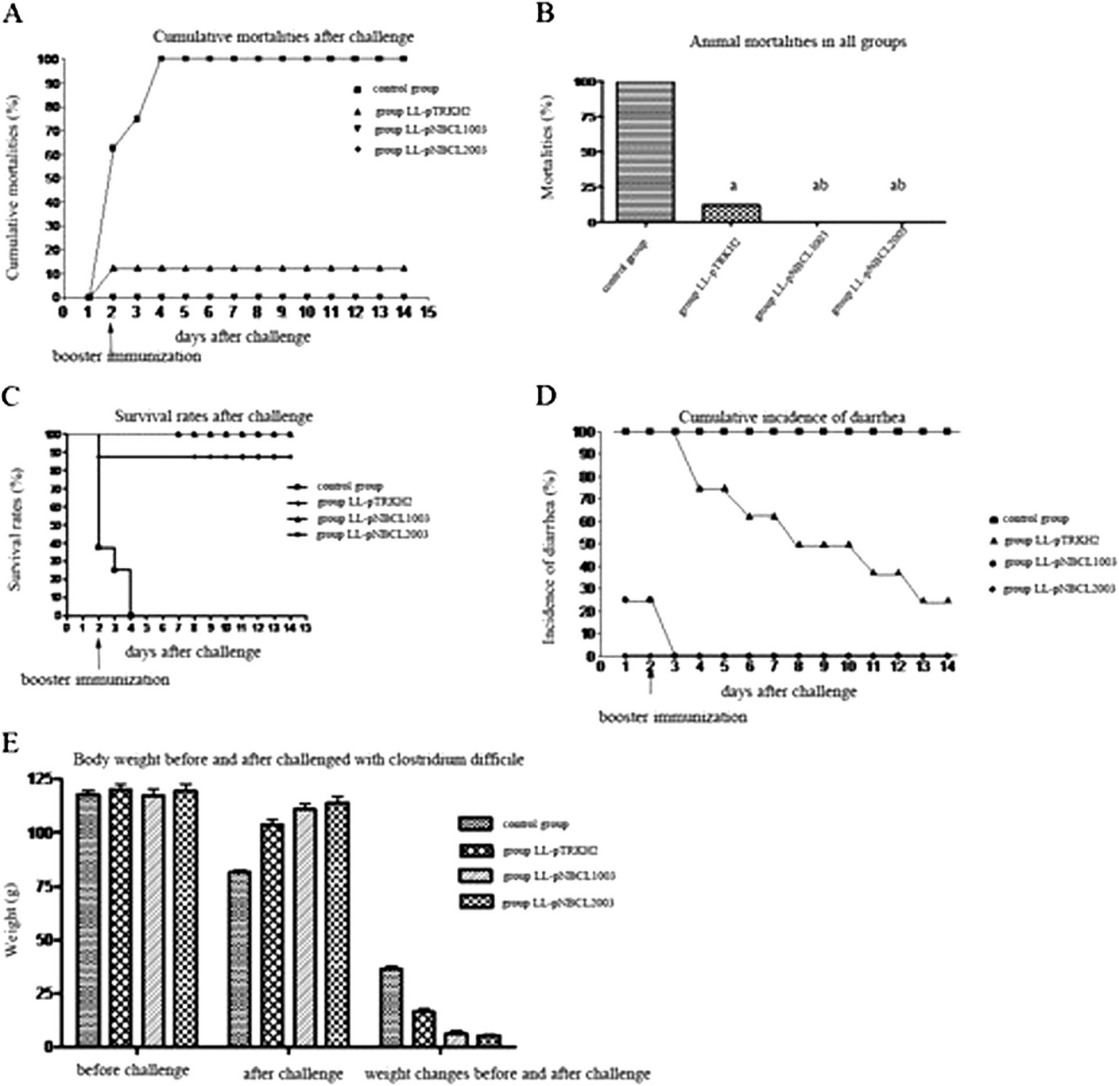
**Effect of recombinant L. lactis vaccine after clostridium difficile challenge. (A)** Cumulative mortalities caused by clindamycin-induced *Clostridium difficile*-associated diseases (CDAD) in different groups of hamsters; **(B)** Mortalities caused by clindamycin-induced CDAD in different groups of hamsters; **(C)** Survival rate (%) in different groups of hamsters; **(D)** Cumulative morbidities of diarrhea caused by clindamycin-induced CDAD in different groups of hamsters; and **(E)** Comparison of body weight in different groups of hamsters before and after challenge with *Clostridium difficile*.

In the control group, all hamsters had light stool, wet perianal area and tail, less activity with response to stimuli, and a soft abdomen on day 1 after the *C. difficile* challenge; few animals had bloody stools as observed by naked eye. The majority of animals in the control group had damp claws and lower abdomen, curved or arched bodies with no activity, soft abdomen, disappeared balance, and shrugged fur on day 2 post-challenge; few animals died. All animals in the control group died by the end of day 4 post-challenge (Figure 2A). In the empty plasmid group, animals subject to *C. difficile* challenge all had light stool and a wet perianal area and tail on day 1 post-challenge; some animals were less active but responded to stimuli; one animal died on day 2 post-challenge (Figure 2A). After *L. lactis* carrying the empty vector was administered again, diarrhea symptoms were alleviated in the infected animals. Till the end of the experiment, two animals still had diarrhea, but their perianal area and tail were not wet. In the secreted-protein plasmid group, two of the animals subject to *C. difficile* challenge had diarrhea on day 1, but their perianal area and tail were not wet. After recombinant *L. lactis* carrying the secreted-protein plasmid was administered again, diarrhea symptoms disappeared in the animals by day 4 post-challenge. In the membrane-anchored group, animals had no symptoms of diarrhea (Table 4).

**Table 4:** **Appearance of hamster in different groups after *Clostridium difficile *challenge (n = 8 each)**

**Manifestations**	**Control group**	**Empty plasmid group**	**Secreted-protein plasmid group**	**Membrane-anchored plasmid group**
Diarrhea	All on day 1	All on day 1	2 on day 1	No
Perianal and tail damp	Yes	Yes	No	No
Paw and abdomen damp	Six on day 2	Only one on day 2	No	No
Activity	Decreased	Decreased	Normal	Normal
Response to stimulation	Decreased, even disappeared	Only one weakened	Normal	Normal
Fur luster	Lackluster	Normal	Normal	Normal
Eating	Decreased	Almost normal	Normal	Normal
Death	All	One	No	No

### Pathological changes in *C. difficile-*infected hamsters

#### Macropathological observations

**Figure 3:**
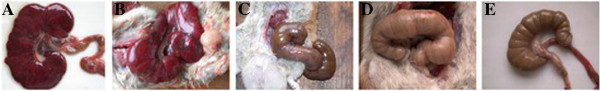
**Changes of intestine loop in all groups at the end of experiment. (A**&**B)** Enlarged and bleeding cecum and upper colon without formed stool in the control group; **(C)** Slightly dilated cecum in the empty plasmid group, with high tension, hydropsia, congestion and no formed stool; and **(D)** Cecum of the secreted-protein and membrane-anchored plasmid groups, with formed stool and no sign of congestion, bleeding, or dilation **(E)**.

**Figure 4:**
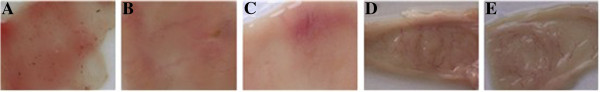
**Macropathological observations in all groups. (A**&**B)** Defective cecal mucosa in the control group, with bleeding, edema and disappearing of vascular lakes. Colon mucosa adjacent to the cecum with edema and ulcer; **(C)** Cecal mucosa of the empty plasmid group, with edema, scattered bleeding points and erosion; **(D)** Cecal mucosa of the secreted-protein plasmid group; and **(E)** Cecal mucosa of the membrane-anchored plasmid group.

After the *C. difficile* challenge, all animals in the control group had cecal expansion and intestinal bleeding on day 2 (Figures 3A and B). Extensive hemorrhagic lesions were observed in the intestine (Figures 4A and B), some of which occurred as mucosal defects. The worst lesions were in the top segments of the cecum and colon. Slight dilation was detected in the small intestine, which was thicker than the colon. The rectum had slight lesions, with no feces observed in the colon. In the empty plasmid group, one animal died on day 2 post-challenge, with a slightly dilated cecum and intestinal mucosa hyperemia (Figure 3C). In its intestinal cavity, a few hemorrhages and obvious congestion and edema were observed within the intestinal mucosa. Interspersed erosion was visible (Figure 4C). Other animals of the empty plasmid group sacrificed at the end of the experiment had a non-dilated cecum and uncongested intestinal mucosa. Animals of the secreted-protein and membrane-anchored plasmid groups had a non-dilated intestine and formed feces in the colon and rectum (Figures 3D and E), with no congestion or edema in the intestinal mucosa (Figures 4D and E).

#### ***Histopathological observations***

**Figure 5:**
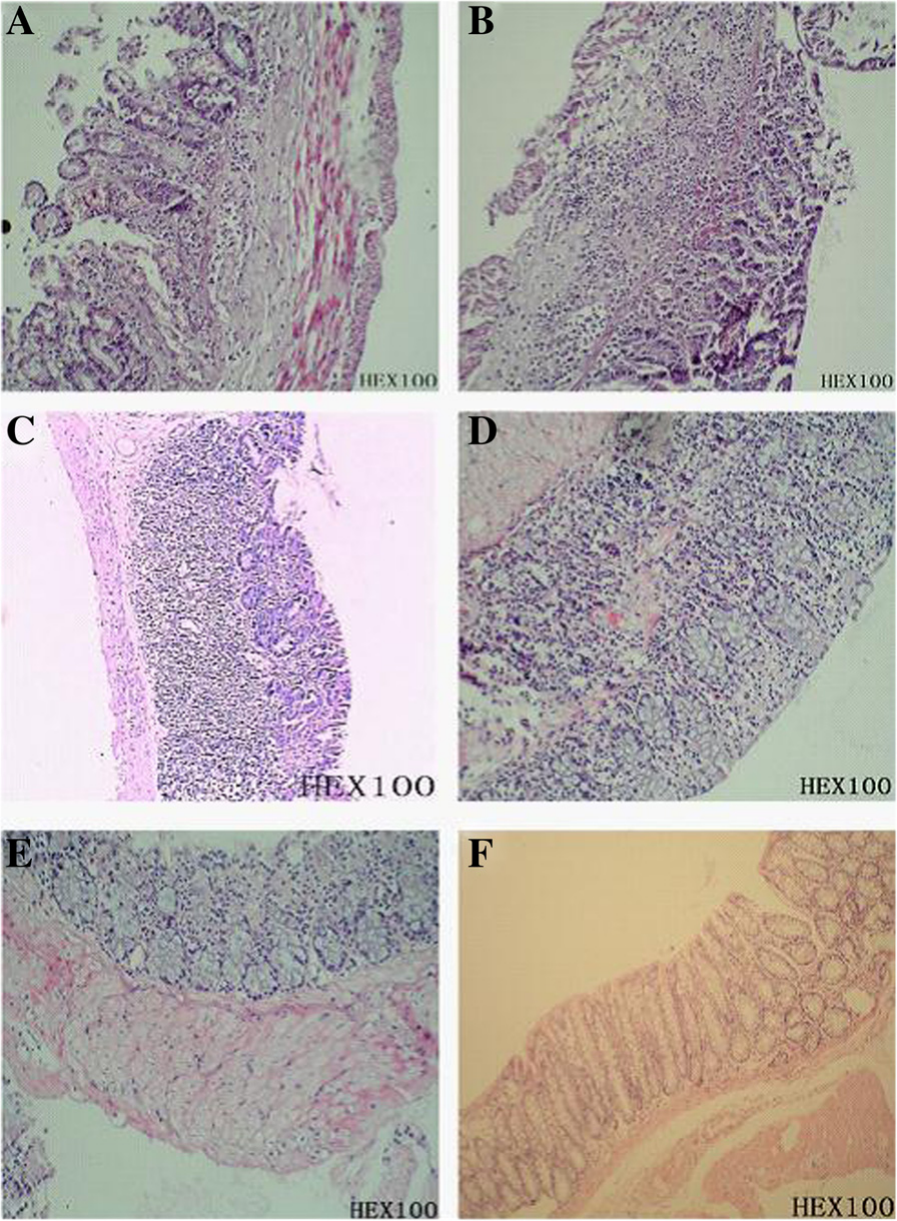
**Histopathological observations in all groups. (A**&**B)** Section of cecal mucosa in the control group. **(C**&**D)** Section of cecal mucosa in the empty plasmid group. **(E)** Section of cecal mucosa in the secreted-protein plasmid group. **(F)** Section of cecal mucosa in the membrane-anchored plasmid group.

**Figure 6:**
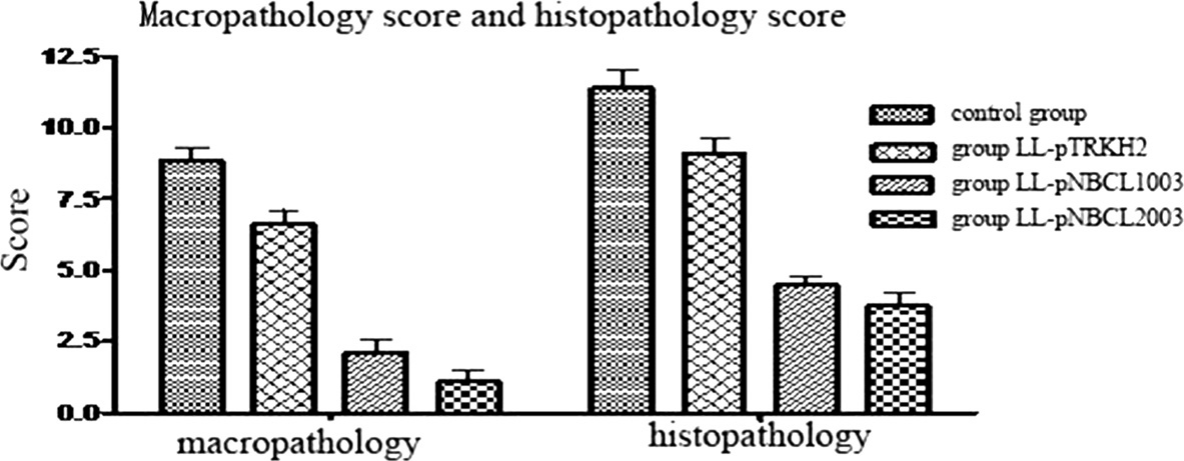
**Comparison of macropathological and histopathological scores among different groups of hamsters.** The macropathological and histopathological scores are higher in the control group than in the 3 immunization groups.

After the *C. difficile* challenge, animals in the control group had mucosal defects, gland destruction, extensive bleeding, submucosal edema, and extensive neutrophil infiltration on day 2 (Figures 5A and B). By comparison, animals in the empty plasmid group had less severe mucosal defect, with a small amount of bleeding and extensive neutrophil infiltration in the submucosa. Moreover, abscess formation was observed (Figures 5C and D). In the secreted-protein plasmid group, animals showed mild damage on the mucosal epithelia post-challenge, with many neutrophils infiltrating the submucosa (Figure 5E). In the membrane-anchored group, the animals had slight defects in mucosa post-challenge, with a few neutrophils infiltrating the submucosa (Figure 5F). Regarding the macropathological and histopathological scores, there are statistically significant differences between the control group and other plasmid treatment groups of hamster (P < 0.005, Figure 6).

### Statistical analysis of evaluation indices

Compared with the control group, the 3 immunization groups had significantly lower mortality rate (P < 0.001, Figure 2B) and higher survival rate/time (P < 0.001, Figure 2C). Of the 3 immunization groups, secreted-protein and membrane-anchored plasmid groups had obviously lower mortality rate than the empty plasmid group (Figure 2B). The incidence of diarrhea was significantly different among all groups (P < 0.001), with the highest level observed in control and empty plasmid groups and the lowest level in the membrane-anchored plasmid group (Figure 2D). As for the weight change from before to after *C. difficile* challenge, the 3 immunization groups had significantly lower values than the control, whereas the secreted-protein and membrane-anchored plasmid groups had significantly lower values than the empty plasmid group (P < 0.001, Figure 2E). There was no significant difference in weight change values between the secreted-protein and membrane-anchored plasmid groups (P > 0.05).

### Occurrence and abundances of *C. difficile* and *L. lactis* in hamster specimens

Prior to the challenge experiment, *C. difficile* was not detected in any feces samples by laboratory cultivation and isolation. However, *C. difficile* was detected in diarrhea stools and intestinal tissues of all animals in the control group after the *C. difficile* challenge. In the empty plasmid group, *C. difficile* was detected in diarrhea stools from all animals 1 day post-challenge, as well as intestinal tissue homogenate of the dead animal 2 days post-challenge. Till the end of the experiment, *C. difficile* was obtained from 3 animals’ stools in the empty plasmid group. In the secreted-protein plasmid group, *C. difficile* was obtained from the stools of two animals, but not in any intestinal tissue specimens. In the membrane-anchored plasmid group, *C. difficile* was detected in the stools of one animal only, but not in its intestinal tissues.

After 1-week administration of CLDM, lactic acid bacteria or bifidobacteria were not isolated from any groups of hamster while fungi were detected in stool samples, suggesting grade III dysbacteriosis (Table 5). To enhance the immunization, *L. lactis* was administered again 2 days after the *C. difficile* challenge. As a result, *L. lactis* was isolated from stools of animals in the 3 immunization groups on day 3 post-challenge. The number of *L. lactis* on day 14 was less than that that detected on day 7 post-challenge. In addition, *L. lactis* was isolated from intestinal mucosa homogenates of the 3 immunization groups, while *C. difficile* was detected in intestinal mucosa homogenates of animals in the empty plasmid group (Table 5).

**Table 5:** **Cultivation and enumeration of *Lactococcus lactis *in cecal mucosa and stool of *Clostridium difficile*-infected hamsters**

**Group (n = 8 each)**	***L. lactis *in cecal mucosa**	***L. lactis *from stool (10**^**6**^**CFU/g)**	
	(10^4^CFU/g)	Day 7	Day 14
Control group	0.413 ± 0.1	Fungi^*^	
Empty plasmid group	104.75 ± 8.41 **ab**	109 ± 6.95 **e**	69.38 ± 6.44dc
Secreted-protein plasmid group	108.25 ± 4.57 **ab**	124.63 ± 7.92 **e**	64.38 ± 6.37dc
Membrane-anchored plasmid group	105 ± 3.9 **ab**	104 ± 3.45 **e**	53.75 ± 4.30dc

**a** compared with control group (F=96.289, P=0.000); **b** compared among immunization groups (F=0.107, P=0.899); **e** compared among immunization groups (F=0.690, P=0.513); **d** compared among immunization groups (F=1.9, P=0.174); and **c** compared between days 7 and 14, P < 0.01. * Grade III dysbacteriosis – appearance of opportunistic flora on top of hemolytic and lactose-negative escherichia, growth of cocci and suppression of lacto- and bifidobacteria (Ladodo scale).

### Anti-TcdA antibody tilter in hamster serum and intestinal fluid

**Figure 7:**
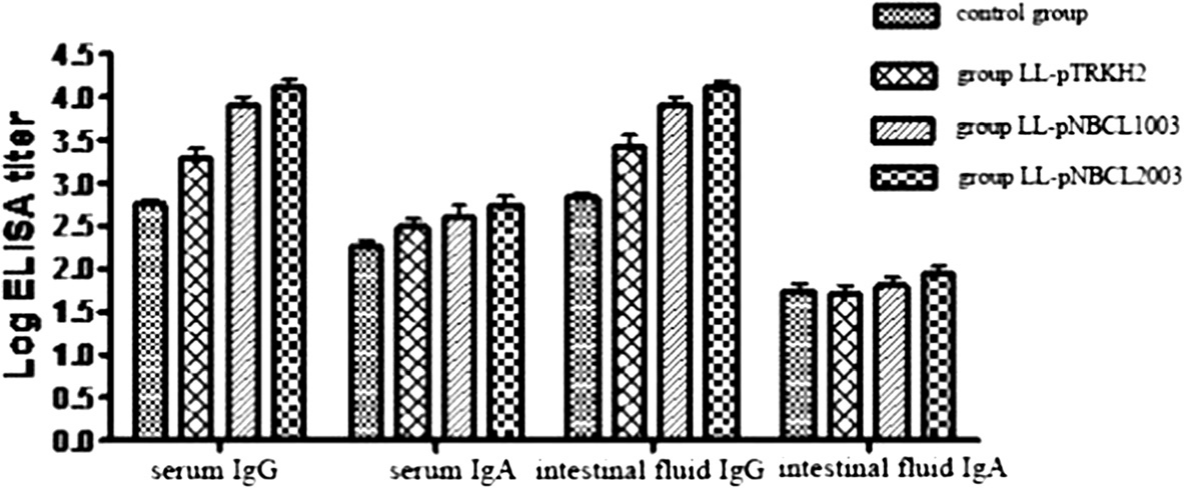
**Anti-toxin A (TcdA) IgG and IgA antibodies in serum and intestinal fluid of different groups of hamsters.** The anti-TcdA IgA titer in serum of secreted-protein and membrane-anchored plasmid groups was higher than that of the control group (P < 0.05). There was no significant difference in anti-TcdA IgA titer of intestinal fluid among all groups.

ELISA assay showed that anti-TcdA IgG and IgA antibodies were produced in serum and intestinal fluid specimens of all animals. In membrane-anchored plasmid group, the titers of IgG and IgA antibodies directed against TcdA were 1.5 × 10^4^ and 6.7 × 10^2^ in the serum, and 1.45 × 10^4^ and 1 × 10^2^ in the intestinal fluid, respectively. In secreted-protein plasmid group, the titers of anti-TcdA IgG and IgA were 9.5 × 10^3^ and 5.6 × 10^2^ in the serum, and 9.4 × 10^3^ and 7.5 × 10^1^ in intestinal fluid, respectively. In the empty plasmid group, the titers of anti-TcdA IgG and IgA were 2.4 × 10^3^ and 3.6 × 10^2^ in the serum, and 3.5 × 10^3^ and 7.5 × 10^1^ in intestinal fluid, respectively. In the control group, the titers of anti-TcdA IgG and IgA were 6 × 10^2^ and 2 × 10^2^ in the serum, and 7.3 × 10^2^ and 6.3 × 10^1^ in intestinal fluid, respectively. Overall, the anti-TcdA IgG titers of hamster serum and intestinal fluid were significantly higher in the secreted-protein and membrane-anchored plasmid groups than in the empty plasmid and control groups (P < 0.001), whereas the anti-TcdA IgA titer of serum was significantly higher in the secreted-protein and membrane-anchored plasmid groups than in the control group (P < 0.05). The anti-TcdA IgA titer of intestinal fluid had no significant differences among all groups (Figure 7).

### Cell morphology as influenced by toxin neutralization

**Figure 8:**
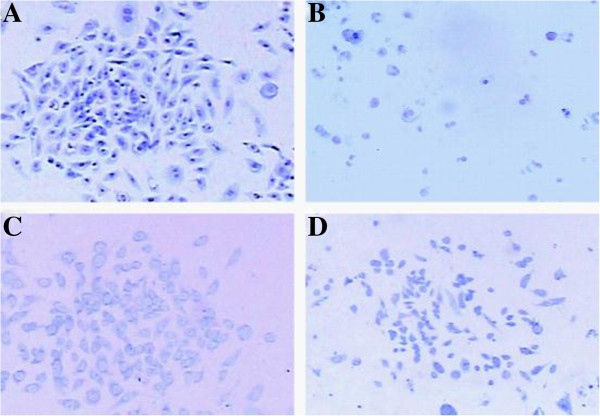
**Results of cytotoxicity neutralization assay. (A)** Control: nucleus was normal. **(B)***Clostridium difficile* toxin A (TcdA) treatment: condensation, fragmentation and dissolution of nuclei were found. **(C)** Anti-TcdA serum pre-treatment: cells became slightly round but still spindly. **(D)** Anti-TcdA serum treatment: cells became round and narrow; nuclei appeared condensed, but some cells were still spindly (×200 magnification).

Light microscopy showed that in the control group, cells were uniform in size and spindle-shaped. In the proliferative phase, cells were round-shaped while the nucleus appeared to be normal (Figure 8A). After TcdA treatment, the cell shape changed from spindly to round while the cell size varied along with nuclear condensation, fragmentation and dissolution (Figure 8B). In the serum pre-incubation group, cells became slightly round with visible spindles (Figure 8C). In the serum treatment group, some cells turned round and narrow and showed nuclear condensation, whereas some cells remained spindly (Figure 8D).

### The effect of anti-TcdA serum on TcdA-induced intestinal epithelial cell apoptosis

**Figure 9:**
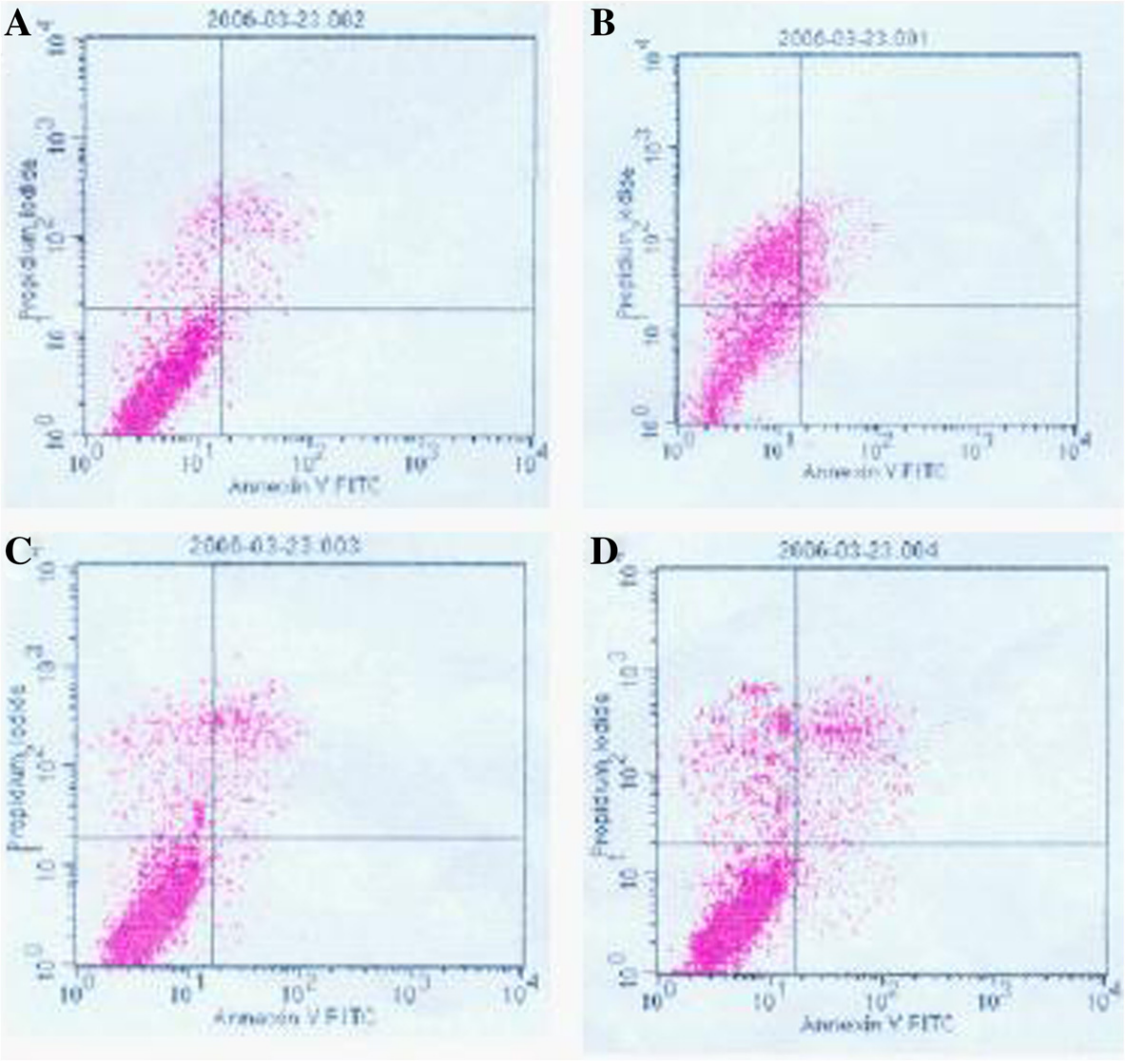
**Effect of anti-*Clostridium difficile *toxin A (TcdA) sera against the cytotoxicity of TcdA assayed by flow cytometry. (A)** Control; **(B)** TcdA treatment; **(C)** Anti-TcdA serum pre-treatment; and **(D)** Anti-TcdA serum treatment.

Flow cytometry showed that a large number of intestinal epithelial cells died after TcdA treatment, with dead and apoptotic cells accounting for 41.59% and 18.34% of the total cells, respectively. By comparison, the proportions of dead and apoptotic cells in total cells were obviously smaller in the other 3 groups, i.e., 7.39%, and 6.47% in the anti-TcdA serum pre-incubation group, 12% and 8.78% in the anti-TcdA serum treatment group, and 3.9% and 3.87% in the control group, respectively. Of these, the proportions of dead and apoptotic cells in total cells were significantly smaller in the anti-TcdA serum pre-incubation group than in the TcdA treatment (χ^2^ = 83.511, P < 0.001) and anti-TcdA serum treatment groups (χ^2^=6.125, P < 0.05) (Figure 9).

### Expression of mucosal inflammatory cytokines in hamster

**Figure 10:**
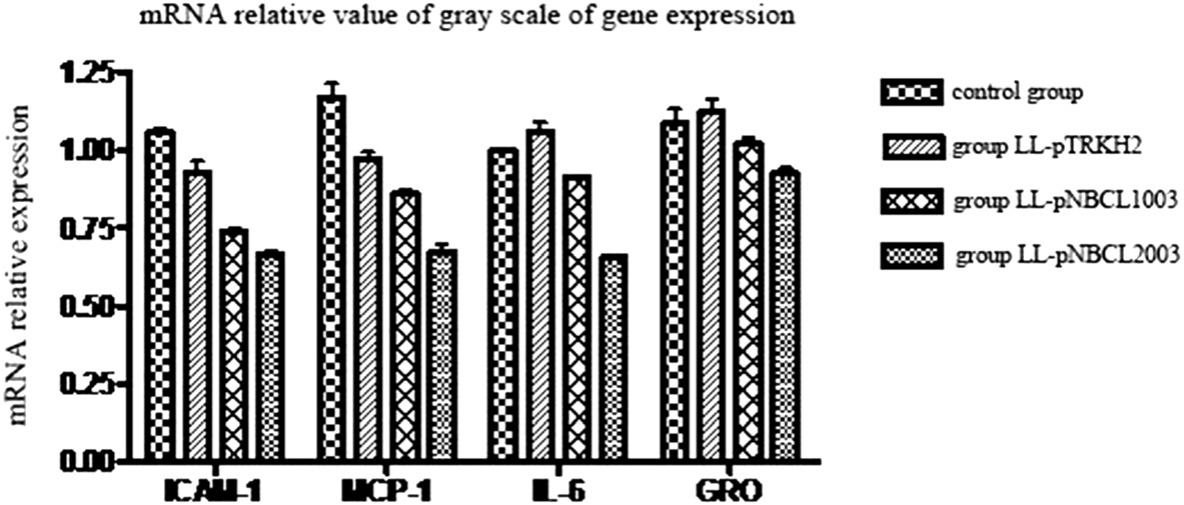
Relative values of the grayscale mRNA levels of mucosal inflammatory cytokines in different groups of hamsters.

PCR assay showed that the mRNA expression levels of mucosal inflammatory cytokines, i.e., ICAM-1, MCP-1, IL-6, and Gro-1 were generally higher in the control and empty plasmid groups than in secreted-protein and membrane-anchored plasmid groups (Figure 10). ICAM-1 and MCP-1 mRNA expression levels varied in similar trends and large ranges, whereas Gro-1 mRNA expression levels hadminor changes among the 4 groups. Overall, the secreted-protein plasmid group had higher ICAM-1, MCP-1, IL-6, and Gro-1 mRNA expression levels than the membrane-anchored group.

## Discussion

### Development of recombinant *L. lactis* live vaccine for *C. difficile*

In accordance with the work by Dieye *et al*. [7], we previously constructed an exogenous gene expression system in *L. lactis*[12], and successfully expressed 14CDTA in the recombinant *L. lactis*[13]. In the present study, we re-constructed the gene expression system by PCR-based gene assembly of the P59 promoter, SPUsp45, 14CDTA containing 12 restriction sites, and the nontoxic adjuvanted TETC, and further developed the recombinant *L. lactis* live vaccine, a live ecological preparation for oral immunization of CDI. As compared to that designed by Dieye *et al*. [7], our modified expression system is more advantageous because it contains an increased number of cloning sites, which are commonly used in gene cloning. Therefore, the modified expression system can be applicable for a variety of bio-engineering purposes. In addition, its immunization effect on CDI animal model is potentially enhanced because of the adjuvant activity of TETC.

### Establishment of CDI animal model in golden hamster

In order to validate the effect of the recombinant *L. lactis* live vaccine for preventing CDI, we established a CDI animal model in golden hamster using CLDM as the inducer. According to Torres *et al.*[3] and Larson *et al.*[17], CLDM is superior to ampicillin, penicillin, and cefuroxime for inducing CDIs in animal models (74 days of susceptible period). In our preliminary experiments, administration of 10^5^CFU *C. difficile* to hamsters after 1-week gavage or intramuscular injection of ceftazidime failed to induce the CDI-associated diarrhea, whereas *C. difficile* challenge after 2-day administration of CLDM resulted in diarrhea symptoms in the animals (data not shown). In addition, we tested SD rats and BALB/c mice as CDI animal models, but neither showed CDI-associated diarrhea symptoms after 1-week administration of CLDM or *C. difficile* (data not shown).

As the live vaccine carried by recombinant *L. lactis* might serve as a probiotic preparation, we performed *C. difficile* challenge on the animals after their intestinal flora were disturbed by 1-week administration of CLDM. Because no *L. lactis* was isolated from animals thereafter, we assume that this procedure could avoid the potential effect of recombinant *L. lactis* live vaccine as a probiotic preparation while simulating clinical infection of *C. difficile* in practice. That is, long-term use of antibiotics causes the disturbance of intestinal flora, negatively affecting the inhibitory factors of *C. difficile* colonization, thereby leading to CDIs. After the *C. difficile* challenge, all animals in the control group suffered severe diarrhea (Table 4), indicating that the CDI model was successfully established in the hamsters.

### Effect of recombinant *L. lactis* live vaccine on *C. difficile-*infected animal model

To evaluate the protective effect of recombinant *L. lactis* live vaccine on *C. difficile-*infected animals, we used the empty shuttle vector carried by *L. lactis* as negative control. After the *C. difficile* challenge, all animals of the empty plasmid group had diarrhea on day 1 (Table 4), suggesting that the intestinal flora were disturbed by continuous administration of CLDM. Compared to the blank control, empty plasmid group had lighter diarrhea symptoms. Despite one died after the *C. difficile* challenge, the diarrhea symptoms were alleviated in the remaining animals, and a few became normal after re-administration of *L. lactis* carrying the empty plasmid. Together these observations indicate that the probiotic *L. lactis* has a protective effect on CDI animal model and may prevent *C. difficile*-associated diarrhea in animals by inhibiting *C. difficile* colonization and/or other mechanisms [18,19].

In the animal group treated with recombinant *L. lactis* carrying secreted-protein plasmid, diarrhea symptoms were found significantly lighter than those in the control and empty plasmid groups (Table 4). Immunological evaluation by ELISA assay showed that the IgG antibody produced in serum and intestinal fluid of secreted-protein and membrane-anchored plasmid groups reacted with TcdA while IgA showed no obvious effect (Figure 7). These observations can be related to previous work by Vaerman *et al.*[20], which indicates that the IgG antibody in serum and intestinal fluid plays a significant role in protecting human and experimental animals from CDI, and that IgA plays aminor role in prevention and control of CDIs. In the present study, none of the animals died in secreted-protein plasmid group after the *C. difficile* challenge. In fact, their diarrhea symptoms were relatively light (Table 4). This is because TcdA acts an enterotoxin as well as a cytotoxin, with a lower cytotoxicity than the *C. difficile* toxin B [19]. Thus, application of TcdA mainly causes intestinal cavity hemorrhage and effusion while preventing the infected animals from death.

In the group vaccinated with recombinant *L. lactis* carrying the membrane-anchored plasmid, no animals had diarrhea symptoms throughout the experiment (Table 4). Pathological analysis and flow cytometry assay confirmed that oral immunization with the recombinant *L. lactis* carrying membrane-anchored plasmid most effectively prevented *C. difficile*-induced diarrhea in the animal model as well as apoptosis in the intestinal epithelial cells (Figures 3, 4, 5, and 9). Compared to other immunization groups, membrane-anchored plasmid group had higher tilter of anti-TcdA IgG antibody in the serum and intestinal fluid (Figure 7), with downregulated expression of mucosal inflammatory cytokines, especially IL-6 (Figure 10). It was possible that the number of exogenous *L. lactis* cells *in vivo* gradually decreased due to phagocytosis by the M cells on the intestinal wall. Because membrane-anchored plasmid anchors the recombinant protein onto the cell wall, phagocytosis of *L. lactis* cells carrying such plasmid can more efficiently deliver the antigen to the immune system than that of secreted-protein plasmid. Consequently, the recombinant *L. lactis* carrying membrane-anchored plasmid is most effective for preventing CDI compared to other immunization treatments. Further investigation is guaranteed to explore molecular mechanism(s) of CDI prevention by the recombinant *L. lactis* live vaccine.

## Conclusion

In this study, we constructed a live Lactococcus lactis vaccine expressing the TETC and 14CDTA, then we proved the effectiveness of the vaccine in a hamster modle. We also found that the recombinant L. lactis carrying membrane-anchored plasmid is more effective for preventing CDI than the recombinant L. lactis carrying the secreted-protein plasmid. Further studies are needed to elucidate the mechanisms of the vaccine for preventing CDI. There is a long way to go for the vaccine using by the human owing to the plasmid containing erythromycin resistant gene.

## Competing interests

There is no financial competing interest to declare in relation to this manuscript.

## Authors’ contributions

XQC designed the research. XQY performed the research, analyzed data, and wrote the paper. YGZ, BJ, DY analyzed data, edited the manuscript. All authors read and approved the final manuscript.

## Pre-publication history

The pre-publication history for this paper can be accessed here:

http://www.biomedcentral.com/1471-230X/13/117/prepub
